# Bayesian adaptive dual control of deep brain stimulation in a computational model of Parkinson’s disease

**DOI:** 10.1371/journal.pcbi.1006606

**Published:** 2018-12-06

**Authors:** Logan L. Grado, Matthew D. Johnson, Theoden I. Netoff

**Affiliations:** Department of Biomedical Engineering, University of Minnesota, Minneapolis, Minnesota, United States of America; University of Connecticut, UNITED STATES

## Abstract

In this paper, we present a novel Bayesian adaptive dual controller (ADC) for autonomously programming deep brain stimulation devices. We evaluated the Bayesian ADC’s performance in the context of reducing beta power in a computational model of Parkinson’s disease, in which it was tasked with finding the set of stimulation parameters which optimally reduced beta power as fast as possible. Here, the Bayesian ADC has dual goals: (a) to minimize beta power by exploiting the best parameters found so far, and (b) to explore the space to find better parameters, thus allowing for better control in the future. The Bayesian ADC is composed of two parts: an inner parameterized feedback stimulator and an outer parameter adjustment loop. The inner loop operates on a short time scale, delivering stimulus based upon the phase and power of the beta oscillation. The outer loop operates on a long time scale, observing the effects of the stimulation parameters and using Bayesian optimization to intelligently select new parameters to minimize the beta power. We show that the Bayesian ADC can efficiently optimize stimulation parameters, and is superior to other optimization algorithms. The Bayesian ADC provides a robust and general framework for tuning stimulation parameters, can be adapted to use any feedback signal, and is applicable across diseases and stimulator designs.

## Author summary

Deep brain stimulation (DBS) is an effective therapy for treating motor symptoms of Parkinson’s disease. However, the clinical success of DBS relies on selecting stimulation parameters that both relieve symptoms while avoiding side effects. Currently, DBS devices are programmed using a laborious trial-and-error process, requiring multiple clinic visits over the course of months. As DBS leads and algorithms become more complex, it will become impossible to select optimal DBS parameters manually. There is a clear need for an intelligent, automated approach to parameter tuning. We present a novel Bayesian adaptive dual controller (ADC), which can autonomously tune stimulation parameters. It uses a feedback signal measured from the patient to quantify the efficacy of a set of stimulation parameters, and uses this information to intelligently find the parameters which work best for each individual patient. The Bayesian ADC has the potential to improve DBS efficacy and reduce clinic visits by efficiently finding the best stimulation parameters.

## Introduction

Deep brain stimulation (DBS) is an effective therapy for treating the motor symptoms of Parkinson’s disease (PD), and is often used to complement dopamine replacement therapy in patients who have progressed to severe stages of PD [1]. The clinical success of DBS relies on selecting stimulation parameters that both relieve symptoms and avoid persistent stimulation-induced side effects. Identifying clinically optimized stimulation settings, or in other words programming the pulse generator, is conducted by a movement disorders specialist through a laborious trial-and-error process. The process involves parsing through several free parameters including electrode configuration, stimulation amplitude, pulse frequency, and pulse width. However, because the programming process is both time-intensive and exhausting for the patient [2, 3], most clinical programming visits focus on a truncated set of four monopolar electrode configurations in which stimulation amplitude is increased for each setting to the point of inducing persistent side effects.

Recent advances in DBS technology have rendered the programming process even more challenging. For instance, directional DBS leads with eight [4] or as many as thirty-two contacts [5] are emerging for clinical use, and new stimulation algorithms are increasing the dimensionality of the programming process, adding additional free parameters [6–12]. As these new technologies become more widely available, programming next-generation DBS systems will no longer be feasible with current trial-and-error approaches [13].

Implantable DBS systems have been designed to deliver stimuli and record the resulting neural responses, thus providing a framework for implementing closed-loop DBS algorithms [14] that can intelligently select the optimal stimulation parameters for each patient at any point in time. Key to the development of a closed-loop DBS strategy is defining a biomarker as feedback for a controller; the biomarker must correlate well with PD symptoms, although it need not be causal. Synchronous activity in the beta range (12-35 Hz) of local field potentials (LFPs) is one possible candidate. While the precise role of beta oscillations in the basal ganglia are under debate, increased beta band activity within the basal ganglia has been associated with anti-kinetic symptoms of PD [15]. Specifically, elevated beta power has been observed in the dorsolateral portion of the subthalamic nucleus (STN) in human patients [16–18] as well as the globus pallidus (GP), but to lesser extent [19–21]. There is also evidence that a reduction in beta power, either by medication [22–24] or DBS [25], correlates with improved UPDRS scores.

**Fig 1:**
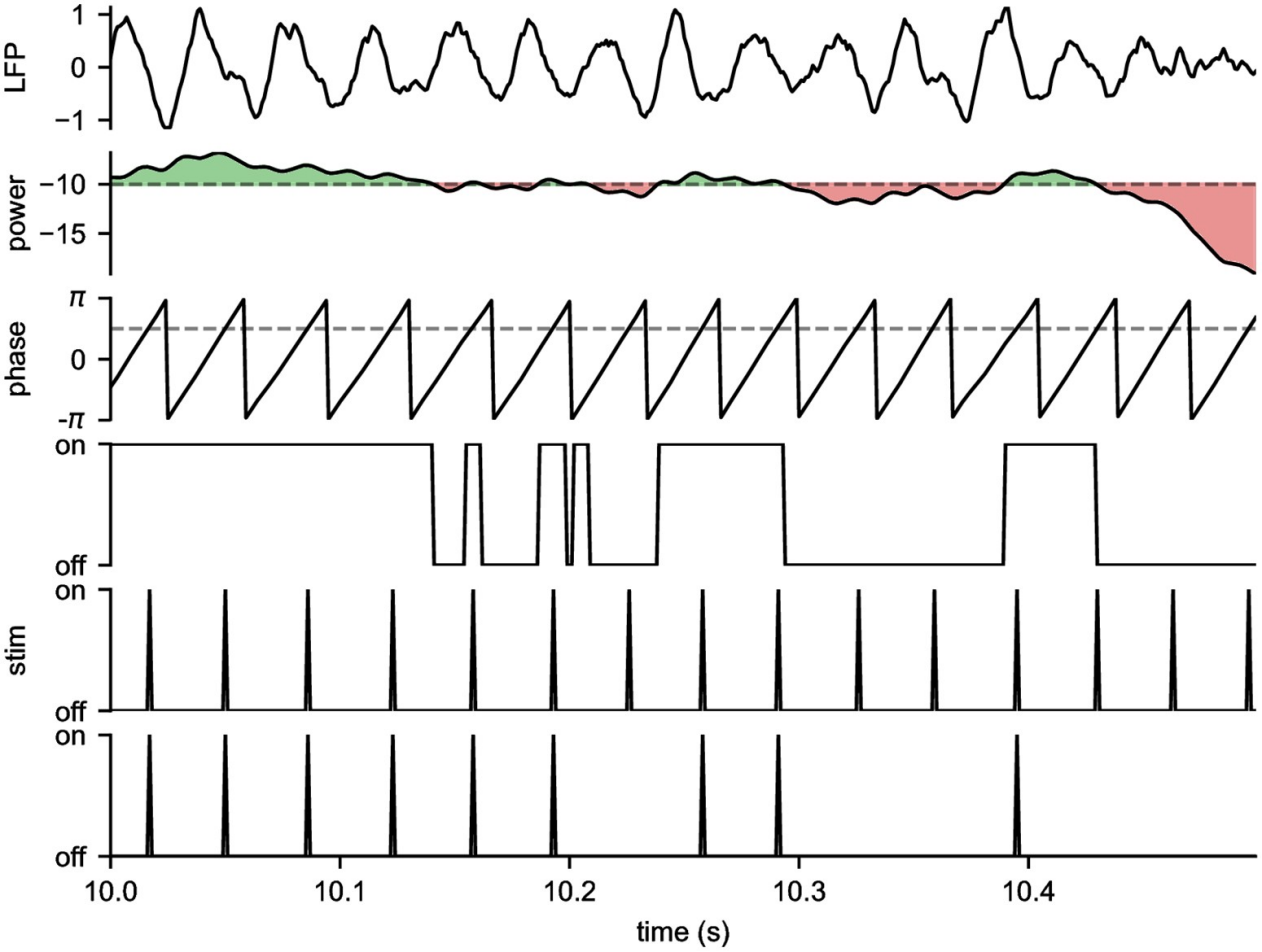
Beta-based feedback stimulation policies. (row 1) Simulated LFP. (row 2, 3) Power and phase calculated from the LFP using the *α*SWIFT algorithm. The dotted lines indicate the manually set power threshold and phase trigger for stimulation. (row 4) Power-based stimulation: high frequency stimulation is turned on when the power is above threshold. (row 5) Phase-based stimulation: individual pulses are delivered when the phase crosses the trigger. (row 6) Combined phase/power-based stimulation: individual pulses are delivered when the phase crosses the trigger, but only if the power is above threshold.

Two separate types of beta-based feedback stimulation policies have been proposed: power or amplitude feedback and phase feedback. In the former implementation, an amplitude-responsive adaptive STN-DBS algorithm initiated stimulation only when the amplitude in the beta band of STN LFPs exceeded a manually set threshold [7, 8]. This approach resulted in significant reduction in parkinsonian motor signs and overall reduction in stimulation on-time compared to conventional, isochronal DBS (cDBS). In the latter case, stimulation was triggered off of the phase of the beta oscillation, delivering phase-locked bursts to optimally disrupt beta oscillations for PD [9, 10] or low frequency oscillations for tremor [11, 12]. However, while both stimulation policies are closed-loop, neither is autonomous; each requires manually setting yet another free parameter. A visualization of these two differing stimulation policies are show in Fig 1, as well as a combined phase and power feedback stimulation policy. We will use the term “power” here, as opposed to “amplitude”, to disambiguate this parameter from other stimulation parameters. As one can readily convert between power and amplitude, the terms are essentially interchangeable.

In this study, we designed and tested a Bayesian adaptive dual control algorithm that can efficiently and autonomously learn the parameters of both phase and power feedback stimulation, as well as other stimulation parameters. We evaluated the algorithm in a computational mean-field model of the basal ganglia-thalamacortical system that simulated beta rhythms and response to electrical stimulation, and we compared the algorithms performance to other optimization strategies.

## Methods

### Computational modeling of the basal ganglia-thalamocortical system

**Fig 2:**
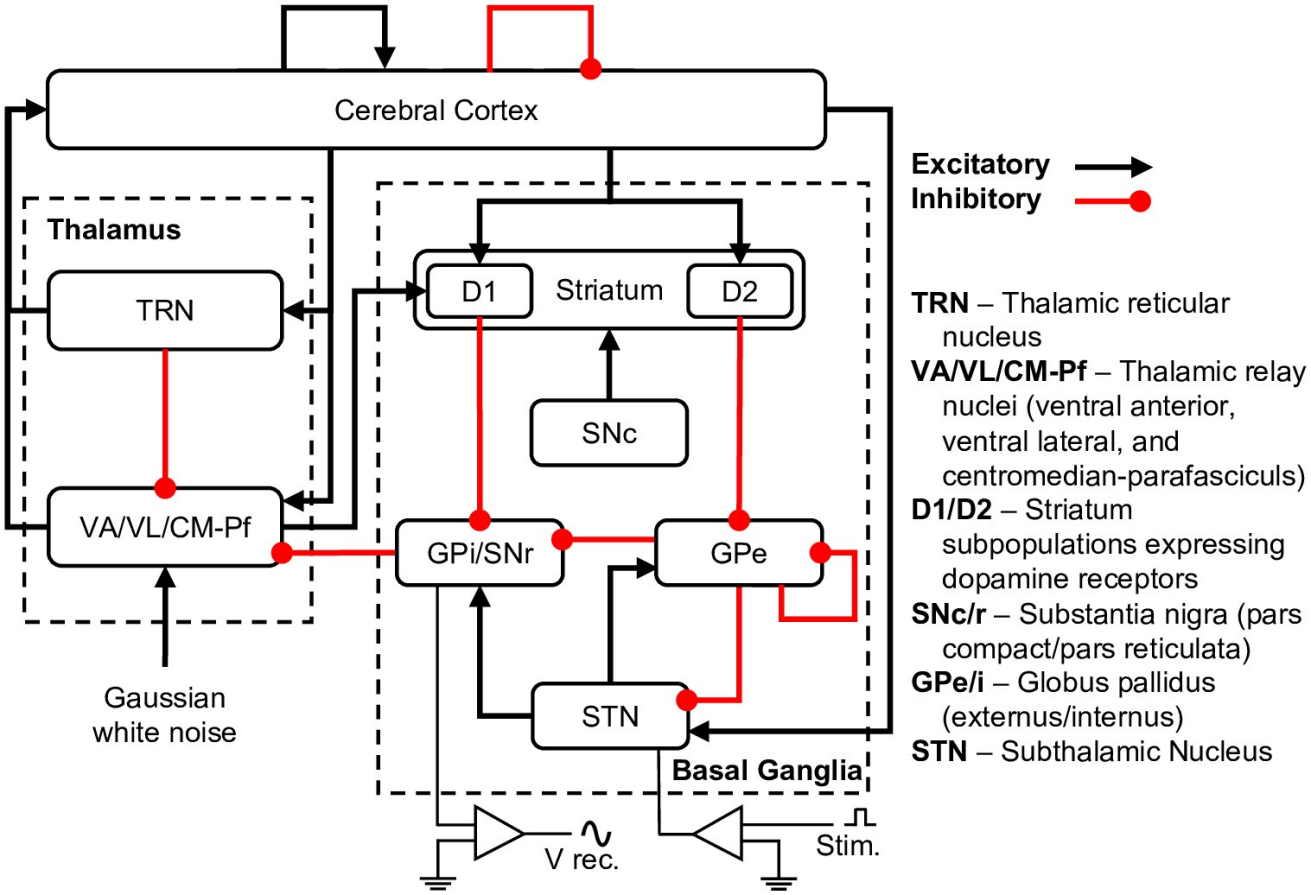
Basal ganglia-thalamocortical system (BGTCS) mean-field model structure. Black arrows represent excitatory connections, red circles represent inhibitory connections. Simulated DBS was applied to the STN, and local field potentials (LFPs) were recorded from the GPi. Adapted from van Albada and Robinson, 2009 [26, 27].

In order to develop and test the adaptive dual control algorithm, we used a physiologically realistic mean-field model of the basal ganglia-thalamocortical system (BGTCS), developed by van Albada and Robinson [26, 27]. The BGTCS modeled the mean firing rate and voltage of nine cortical and subcortical structures with second-order differential equations, the structure of which is shown below in Fig 2. The model was capable of simulating both the naïve state, as well as a dopamine-depleted (DD) state, with a strong beta rhythm. In this study we tested the Bayesian adaptive dual controller in the dopamine-depleted state of the model to suppress its beta oscillation. For a detailed description of the equations governing the model and how parameters were set, see van Albada and Robinson, 2009 [26, 27]. The BGTCS model produced LFP signals generally comparable in spectral content to those measured in humans with Parkinson’s disease undergoing DBS surgery.

**Fig 3:**
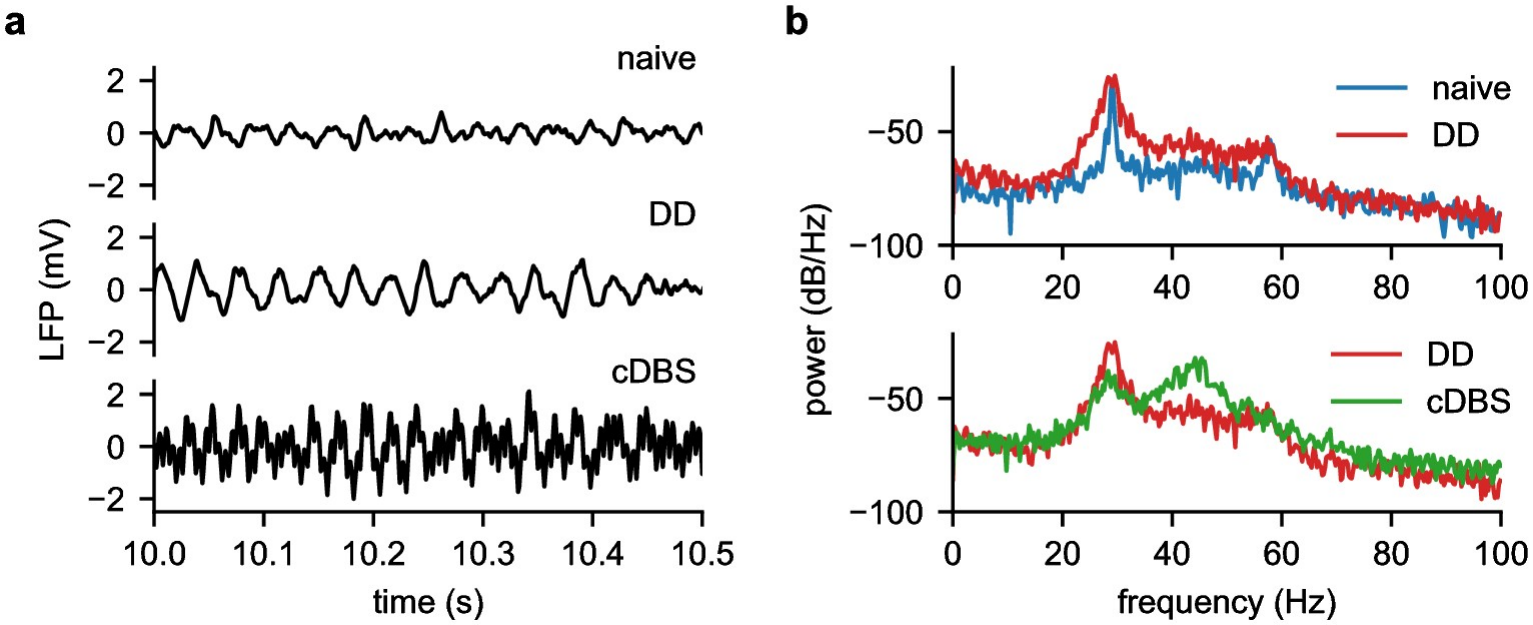
Example BGTCS results. (a) time-series data and (b) PSD analysis in three conditions: naïve, DD, and DD with cDBS in the STN. The model produced a spectral peak at 29 Hz, which increased and widened in the DD state. When cDBS was applied to the STN of the model, the spectral power in beta band decreased.

In order to simulate the effects of DBS within the model, stimuli were incorporated as a direct current injection into the target structure. As the integration timestep of the model (1 ms) was much greater than the duration of the first phase of a typical DBS pulse (60 μs–240 μs), the stimulus pulse was integrated to obtain the total charge, which was then divided by the membrane capacitance to yield the change in voltage due to a single DBS pulse. The resultant ΔV was added directly to the voltage of the target structure. Fig 3 shows example voltage traces from the GPi of the BGTCS in the naïve, DD, and DD with cDBS states, as well as the power spectrum from each trace.

The power spectra revealed several salient features of the BGTCS. First, it produced an oscillation in the beta range (at 29 Hz), and the power of that oscillation increased in the DD state. Second, simulated conventional DBS at 130Hz (cDBS), similar to what has been used clinically, reduced the power of the 29 Hz oscillation. *Thus, the model of the dopamine-depleted state 1) produced oscillations with a pronounced beta peak, and 2) responded to cDBS in a realistic manner*. This model was then used to design, test, and evaluate the Bayesian adaptive dual controller.

### Adaptive dual control for DBS

The tuning of stimulation parameters for DBS was formulated as a control problem: We have a system (the patient) whose symptoms we wish to control (i.e. reduce) with stimulation. However, unlike normal control problems, here we have *dual goals*: We wish to control the patient’s symptoms as well as possible using the best known stimulation parameters, but also must explore the parameter space to identify new parameters that may be better than the current best, thus allowing for better control in the future. This balance between control and information gathering, or *exploitation* and *exploration* leads to the concept of *dual control* [28].

In order to accomplish these conflicting goals, we implemented an *adaptive dual controller* (ADC) for DBS, which is composed of two components: (1) an inner parameterized stimulator and (2) an outer parameter adjustment loop. The inner loop can be any stimulator with parameters to tune, from a traditional cDBS system to new closed-loop DBS algorithms, and may or may not incorporate feedback from the patient. For example, a power-based DBS algorithm would turn stimulation on or off based upon the power of an oscillation measured from the patient. Conversely, cDBS would not measure any feedback signals.

**Fig 4:**
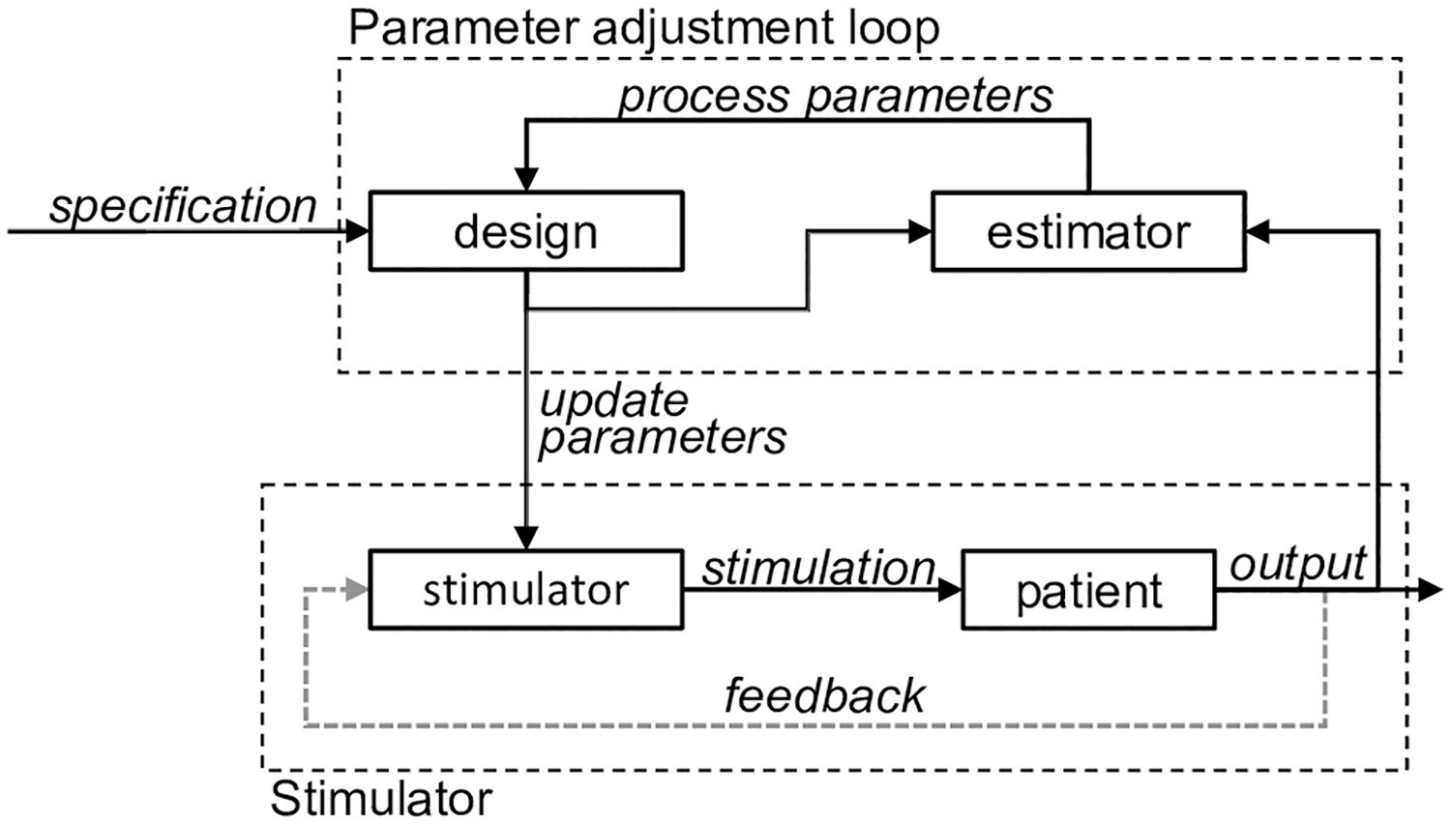
Adaptive dual controller (ADC) for DBS. The ADC has dual goals (exploitation and exploration), and is composed of two loops: an inner parameterized stimulator and an outer parameter adjustment loop. The inner loop may incorporate feedback from the patient to alter stimulation. The outer loop is composed of an estimator and a design block, and is given a specification. The estimator builds a model of the relationship between stimulation parameters and some measure of patient outcome, which it passes on to the design block. The design block then incorporates this information with the specification to select new parameters for the inner loop. The inner loop operates on a much shorter timescale than the outer loop.

The outer parameter adjustment loop acts to tune the parameters of the stimulator, and operates on a relatively long timescale. The outer loop is given a specification, or goal, which it attempts to meet through an iterative process: selecting a parameter value (or values), observing the effect of that value on its goal, estimating the effects of new values, and then selecting the next value. For example, with an power-based DBS algorithm, the outer loop would begin by selecting a power threshold for the inner loop. The inner loop would then execute stimulation with that parameter value for some pre-determined amount of time, after which the outer loop would observe the effects of that value on some biomarker and select a new value. The general structure of an adaptive dual controller for DBS is shown in Fig 4.

Traditional cDBS can be viewed as a simplistic ADC, where an isochronal stimulator takes the place of the parameterized stimulator, and the clinician acts as the parameter adjuster. The clinician’s specification is to improve the patients quality of life. During a clinic visit, they select stimulation parameters and observe the effects. The clinician uses his or her experience to build a mental estimation of the relationship between parameters and quality of life, and uses this map to intelligently determine which parameter combinations to try. At the end of the visit, however, the loop is broken and the patient is sent home with the clinician-optimized settings.

Here, we designed a Bayesian ADC with two components: an inner phase/power feedback stimulator, and an outer Bayesian optimization parameter adjustment loop. We first describe the components individually, and then describe the combined Bayesian ADC.

### Inner loop—Real-time phase/power feedback stimulation

The inner feedback stimulator had three parameters: (1) oscillation phase trigger, (2) oscillation power threshold, and (3) stimulus amplitude. In order to implement phase and power feedback stimulation, a real-time method of accurately estimating both the phase and power of an oscillation was paramount. Previously, phasic stimulation had been accomplished by band-pass filtering the signal and then using the time since the preceding zero crossing to approximate phase [12]. Power-based stimulation had been achieved by rectifying and smoothing the band-passed signal for 400 ms [7, 8]. The Hilbert transform is often used to extract the phase and power of a signal. However, the Hilbert transform is acausal, making it impossible to implement in real time.

We recently developed a novel sliding Fourier transform, called the Sliding Windowed Infinite Fourier Transform (SWIFT)

$$X_{n}\left(\omega \right)=e^{-1/\tau }e^{j\omega }X_{n-1}\left(\omega \right)+x\left[n\right],$$ (1)

along with the *α*SWIFT,

$$X_{n}{\left(\omega \right)}_{\alpha }=X_{n}{\left(\omega \right)}_{slow}-X_{n}{\left(\omega \right)}_{fast},$$ (2)

described in [29]. Unlike other methods of phase/power estimation, the SWIFT directly and efficiently calculates the Fourier transform of the signal in real time, centered on *ω* = 2*πf*/*f*_*s*_ and windowed with an infinite length, causal exponential window. In fact, the SWIFT is a causal approximation of the Hilbert transform. The *α*SWIFT employs the *α* window (the difference between two exponentials with different time constants), and has improved frequency resolution. Here, we used the *α*SWIFT to calculate the phase and power of the beta oscillation in real time.

The SWIFT has two parameters which control its behavior: the center frequency *ω*, and the time constant *τ* (or two time constants, *τ*_*slow*_ and *τ*_*fast*_ for the *α*SWIFT). The center frequency, *ω* was set to match the center frequency of the beta peak in the model. The (slow) time constant controls the time-frequency tradeoff of the SWIFT: a shorter time constant leads to higher temporal resolution, but lower frequency resolution (wider frequency response). To balance this tradeoff, we matched the width of the SWIFT’s frequency response to the width of the model’s beta peak at -6 dB (or 50% power reduction). The model’s beta peak had a width of ±1.15 Hz at -6 dB, and so we set *τ*_*slow*_ = 0.240 s to match, which can be readily calculated from the Fourier transform of the SWIFT’s exponential window. *τ*_*fast*_ was set to *τ*_*slow*_/5, which smooths the output without significantly altering the SWIFT’s frequency response. Fig 1 shows the phase/power feedback stimulation algorithm operating on an example LFP, extracting phase/power using the *α*SWIFT, and triggering stimulation off phase when the power is above threshold.

In this context, the SWIFTs parameters are selected to filter the signal around the oscillation produced by the BGTCS. The SWIFT parameters for a physiological signal could be selected in a similar manner: The center frequency and width can be estimated from the power spectral density measured from a sample signal. A concern is that a physiological signal’s center frequency may wander more than the BGTCS model; this could be addressed by periodically re-estimating the SWIFT parameters from the raw signal. Alternatively, Jackson et al, 2016 described a method of estimating the real time phase of a frequency-modulated signal by combining three real time Fourier transforms (RTFT) operating at neighboring frequencies, which produces a flat frequency response over the frequency band of interest. Their method could easily be augmented to use the SWIFT in the place of the RTFT [30].

### Outer loop—Bayesian optimization of stimulation parameters

While many optimization algorithms could be used for the outer loop, the problem of creating an ADC for DBS has several constraints which make Bayesian optimization (BayesOpt) ideal. The goal of BayesOpt is to find the minimum of the objective function with as few evaluations as possible [31–34], and indeed is among the most efficient algorithms at doing so [32, 35–38]. BayesOpt also provides a framework for explicitly balancing exploration and exploitation in order to efficiently find the global minimum. To reduce the number of function evaluations, BayesOpt only approximates the objective function accurately in regions where it is profitable to do so, and samples coarsely everywhere else [39]. This is ideal for tuning stimulation parameters as the patient is likely to have little tolerance for exploration, and so we wish to find their optimal settings with as few steps as possible.

The power and efficiency of BayesOpt stems from the incorporation of prior belief about the objective function with available evidence (through Bayes theorem) to build a model of the objective function,

P(M|E)∝P(E|M)P(M). (3)

That is, the *posterior* probability of a model *M* given some evidence *E*, is proportional to the *likelihood* of *E* given *M* multiplied by the *prior* probability of *M*. BayesOpt then uses this model to direct sampling and trade off exploration and exploitation [40].

BayesOpt consists of three steps. First, a prior distribution is defined over the objective function. Second, a set of *N* previously gathered measurements, $D_{1:N}$, are combined with the prior through Bayes rule to obtain a posterior distribution. Finally, the acquisition function, which is a function of the posterior distribution that predicts the utility of sampling, is used to determine where next to sample to maximize the utility.

#### Defining the prior

First, we place a prior distribution over the objective function, *f*(*θ*). In our case, the objective function was the mean beta power (measured over several seconds), and was a function of the stimulation parameters, *θ*. While many models can be used as the prior, Gaussian process (GP) priors are favored and are well suited as they satisfy the “simple and natural” conditions: (i) continuity of the objective function *f*(*θ*), (ii) homogeneity of the prior *P*, and (iii), independence of *m*^*th*^ differences [35].

$$f\left(\theta \right)∼GP(m\left(\theta \right),k\left(\theta ,\theta^{\prime }\right)).$$ (4)

A GP can be thought of as a distribution over functions, completely specified by its mean function, *m*(*θ*), and its covariance function (often referred to as the kernel), *k*(*θ*, *θ*′), which computes the “similarity” of any two points, *θ* and *θ*′. Instead of returning a single value at each point in the parameter space, the GP returns two values: the mean and variance of a normal distribution. The prior mean is often assumed to be zero everywhere, *m*(*θ*) = 0, although in our case we learn the mean function as the mean of the training data. The prior covariance matrix is computed using a kernel between the inputs. We used the Matern kernel, which we modified to be periodic in phase. The parameters of the kernel (length scale and noise level) were learned by maximum a posteriori (MAP) estimation.

#### Computing the posterior

We then compute the posterior distribution by combining a set of *n* previously gathered measurements, $D_{1:n}={\left\{\theta_{i},f\left(\theta_{i}\right)\right\}}_{i=1}^{n}$, with the prior through Bayes rule. Let us denote the value of the function at the arbitrary point *θ*_*i*_ as *f*_*i*_ = *f*(*θ*_*i*_), and the vector of previous points as **f**_1:*n*_ = [*f*_1_, …, *f*_*n*_]^*T*^. The formula for the predictive distribution can be readily derived as

$$P\left(f_{n+1}|D_{1:n},\theta_{n+1}\right)=N(\mu_{n}\left(\theta_{n+1}\right),\sigma_{n}^{2}\left(\theta_{n+1}\right)),$$ (5)

where

$$\mu_{n}=k^{T}K^{-1}f_{1:n},$$ (6)

$$\sigma_{n}^{2}=k\left(\theta_{n+1},\theta_{n+1}\right)-k^{T}K^{-1}k,$$ (7)

are the mean and variance of the posterior distribution [41]. Here **k** denotes the vector of kernels *k*(*θ*_*n*+1_, *θ*_*i*_) for *i* = 1, …, *n*, and **K** is the full kernel matrix of *θ*_1:*n*_ whose *ij*^*th*^ entry is given by *k*(*θ*_*i*_, *θ*_*j*_) for *i* = 1, …, *n* and *j* = 1, …, *n*.

#### Minimizing the acquisition function

Finally, BayesOpt directs where next to sample by minimizing the *acquisition function*, *u*(*θ*). The acquisition function serves to guide the search to the optimum by modeling the expected utility of sampling at *θ*_*n*+1_. Typical acquisition functions achieve low values in regions where either the predicted mean is low, the uncertainty is high, or both. We chose to use the Gaussian process lower confidence bound (GP-LCB) acquisition function:

$$\text{GP}-\text{LCB}\left(\theta_{n+1}\right)=\mu_{n}\left(\theta_{n+1}\right)-\kappa \sigma_{n}\left(\theta_{n+1}\right),$$ (8)

where *κ* ≥ 0. BayesOpt thus selects the next evaluation point, *θ*_*n*+1_, by minimizing the acquisition function, e.g. sampling at $\text{arg}\;{\text{min}}_{\theta_{n+1}}u\left(\theta_{n+1}|D_{1:n}\right)$. The acquisition function also governs the trade-off between exploration and exploitation. In GP-LCB, the *κ* parameter determines the exploration-exploitation trade-off; high *κ* encourages exploration, while a low *κ* encourages exploitation. With $\kappa_{n}=\sqrt{\nu \tau_{n}}$, *ν* = 1, and *τ*_*n*_ = 2 log(*n*^*d*/2+2^*π*^2^/3*δ*), it can be shown that this method is *no regret* with high probability. For a full description and proof, see Srinivas et al., 2010 [42]. However, in our situation we chose to favor exploitation, and so set *v* = 0.25.

#### Bayesian optimization example

**Fig 5:**
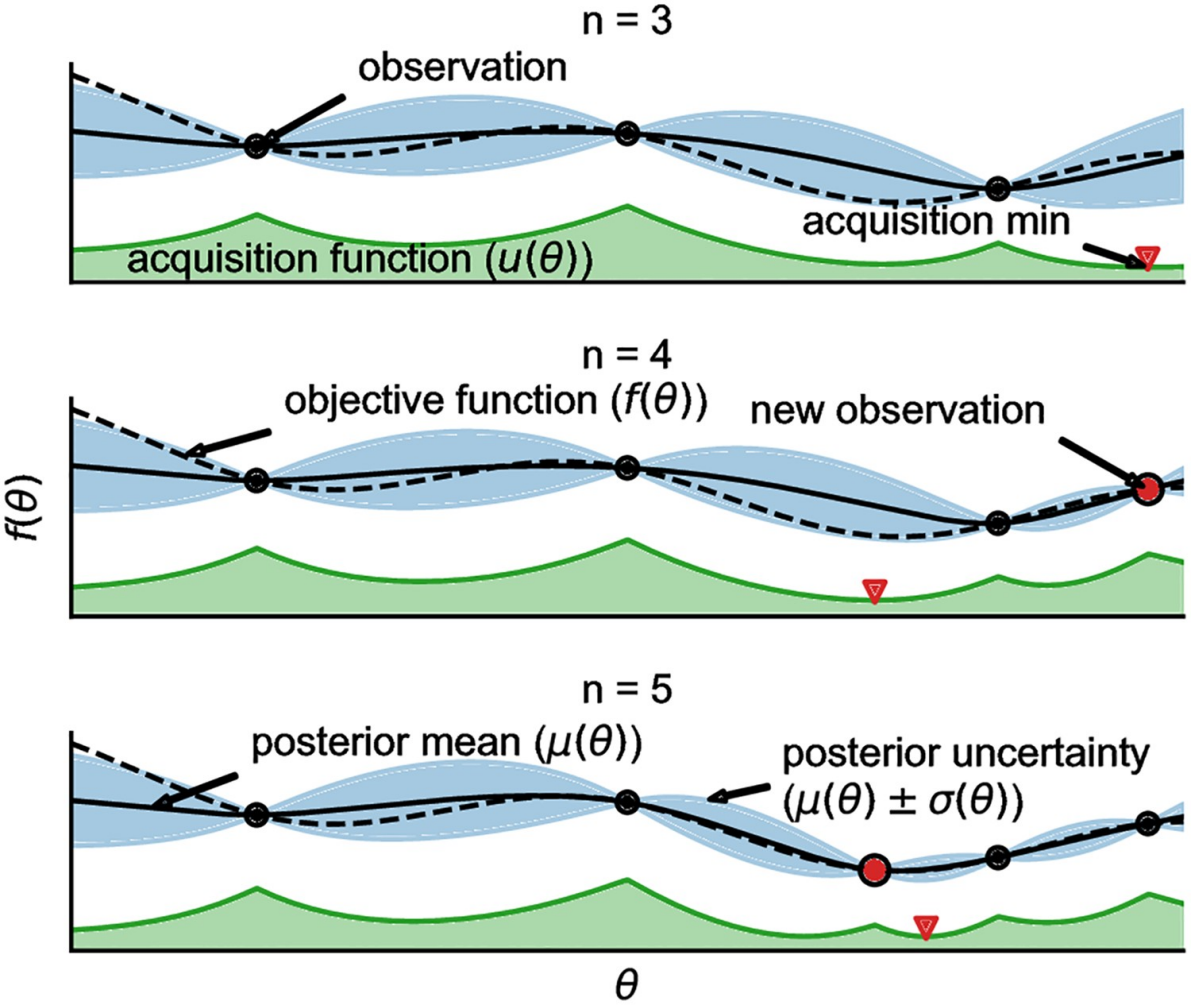
Bayesian optimization example. Three iterations of Bayesian optimization minimizing a 1D function. The figure shows a Gaussian process (GP) approximation (solid black line and blue shaded region) of the underlying objective function (dotted black line). The figure also shows the acquisition function (green). The acquisition function (GP-LCB) is the difference of the mean and variance of the GP (multiplied by a constant), which Bayesian optimization minimizes to determine where to sample next.

Fig 5 shows a typical run of BayesOpt on a 1D problem. The optimization started with 3 points, from which it fitted a Gaussian process (GP). BayesOpt then computed an acquisition function from the GP (which incorporated both the mean and variance of the GP to model the utility of sampling) and minimized it to determine where to sample next. Finally, the objective function was sampled at the new point, and the process was repeated. For a detailed description of Bayesian optimization, see Brochu, Cora, & Freitas, 2010, and Rasmussen & Williams, 2004 [40, 41].

### Bayesian adaptive dual controller

Putting the above components together, we constructed a Bayesian adaptive dual controller. The controller had two components: an inner feedback stimulation loop, which applied stimulation based on the phase/power of the beta oscillation, and an outer Bayesian parameter adjustment loop which optimized the parameters of the inner feedback stimulator to maximally suppress the beta oscillation.

#### Inner loop

The inner loop of the Bayesian ADC was composed of a closed-loop phase/power feedback stimulator. The stimulator measured the LFP from the globus pallidus internus (GPi) of the BGTCS (Fig 2), and triggered stimulation off of the phase and power of the beta oscillation, estimated in real time using the *α*SWIFT (Fig 1). The inner loop had three parameters: (1) oscillation phase trigger, (2) oscillation power threshold, and (3) stimulus amplitude, which were optimized by the outer loop. The inner loop operated on a timescale of 1 ms, the same as the BGTCS.

#### Outer loop

**Fig 6:**
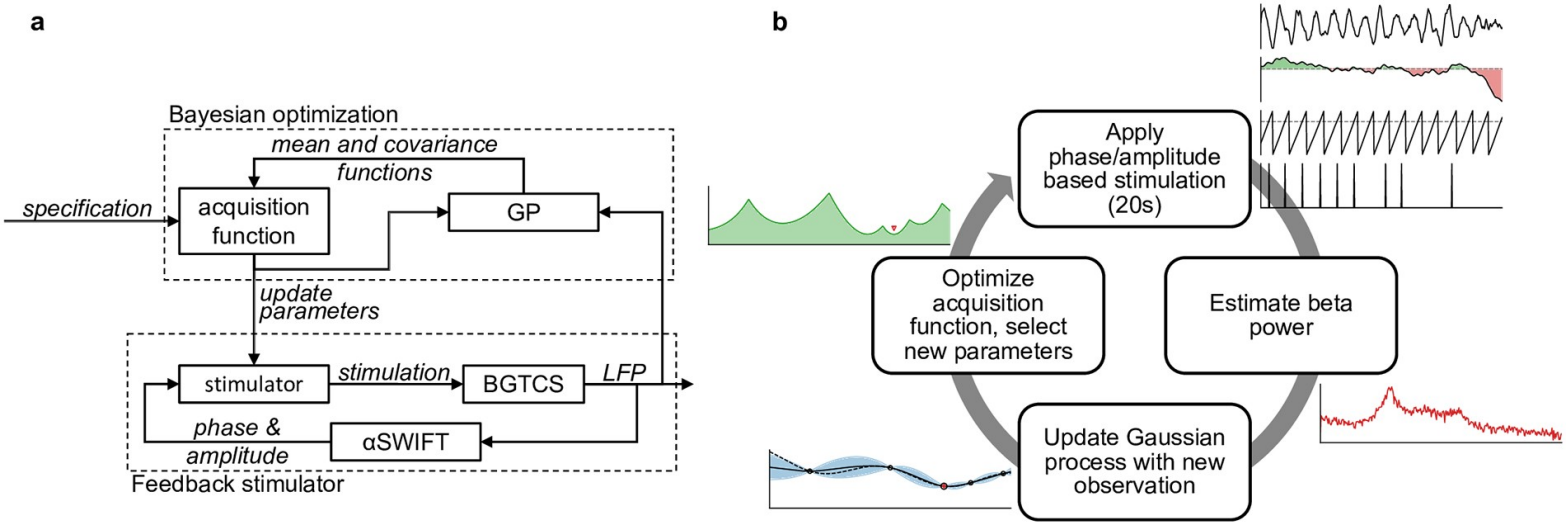
Overview of the Bayesian ADC. (a) Bayesian ADC control diagram. The Bayesian ADC’s inner loop was composed of a phase/power based feedback stimulator. The outer Bayesian optimization loop was composed of a Gaussian process (GP), and acquisition function. The Gaussian process builds a model of how the stimulation parameters affect the feedback signal, and the acquisition function uses this information to select the next parameter set. (b) Overview of the Bayesian ADC’s cyclic operation. The Bayesian ADC sets the stimulator parameters and applies phase/power based stimulation to the BGTCS for 20s. It then estimates the effect of those parameters on beta power, and updates its GP with the new observation. Finally, it optimizes its acquisition function, and selects the next parameter set.

The outer loop of the Bayesian ADC employed Bayesian optimization to intelligently sample the parameter space and select the optimal set of parameters. The outer loop operated on a timescale of 20 s, much longer than the inner loop. After selecting a new parameter set, the outer loop would wait 10 s, which allowed the BGTCS to settle into a steady state. The outer loop would then estimate the power of the beta oscillation over the next 10 s by keeping a running average of the oscillation power. It then would update its internal Gaussian process with the new observation, minimize its acquisition function, and select the next parameters to sample. The Bayesian ADC’s control diagram is shown in Fig 6a, and an overview of how the Bayesian ADC functions is shown in Fig 6b.

## Results

The Bayesian ADC was tested in the BGTCS. First, we show that the BGTCS responded differentially across the 3D parameter space of the feedback stimulator, and that there existed a minimum. Next, we present a 1D example of the Bayesian ADC optimizing stimulus phase trigger in the model. Finally, we show that the Bayesian ADC converged quickly to the global minimum in all cases, and compared the Bayesian approach to other standard optimization methods.

### Parameter sweep

**Fig 7:**
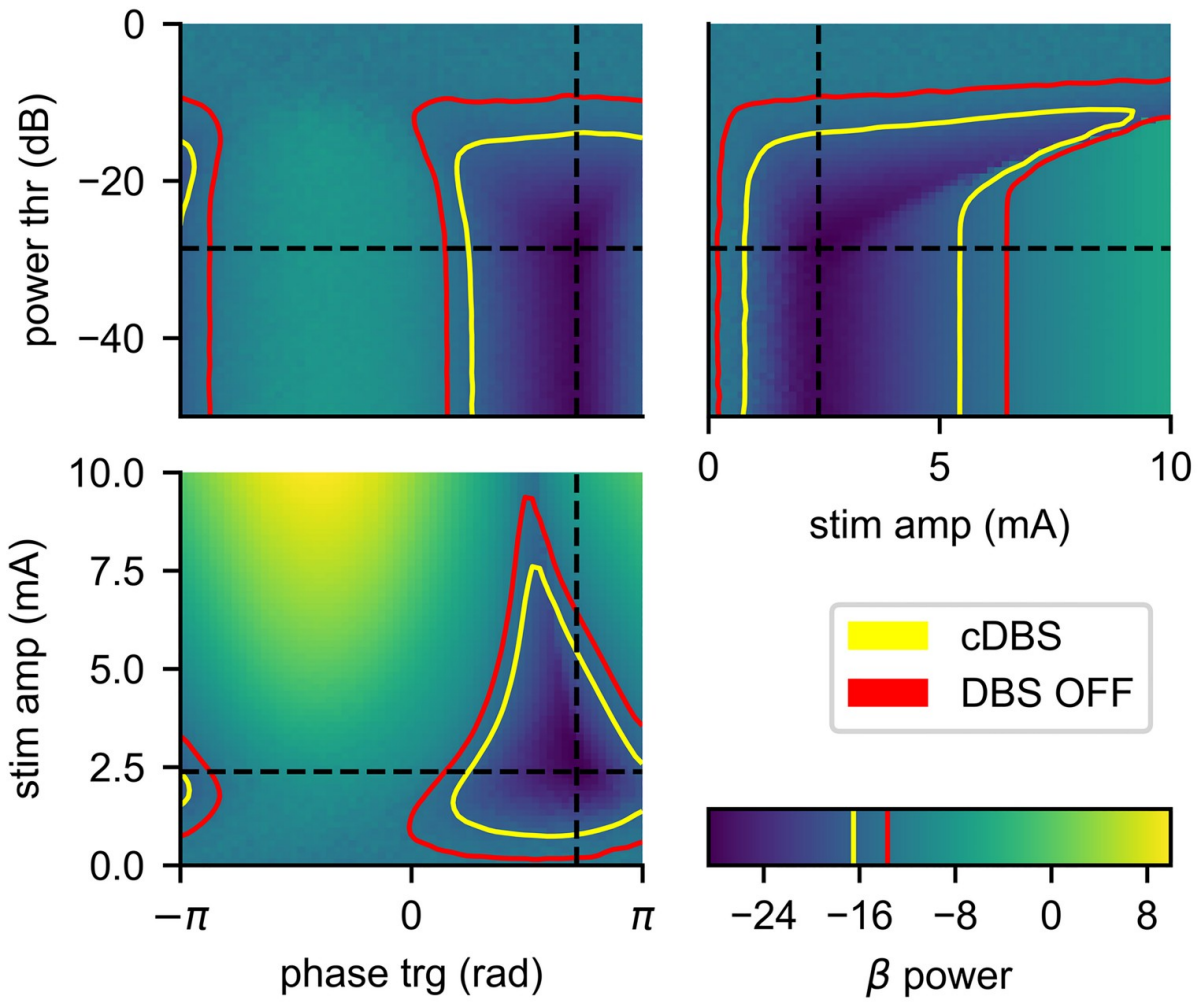
Beta power as a function of stimulation parameters. Feedback stimulator parameter sweep over stimulus phase trigger, power threshold, and amplitude. The sweep revealed a global minimum of -28.6 dB at 〈2.24 rad, 2.37 mA, -28.6 dB〉, denoted with dashed black lines. The sweep revealed a complex underlying landscape with flat regions (in response to power threshold), nonlinearities (in response to stimulation amplitude), and shallow local minima (high power thresholds). The red and yellow lines indicate the isoclines of the beta power with DBS OFF and cDBS, respectively.

In order for the Bayesian ADC to find an optimal parameter set, there must exist at least one minimum over the feedback stimulator’s parameter space. We swept the space on a 64^3^ grid (oscillation phase trigger, oscillation power threshold, and stimulus amplitude), and measured the average beta power over the last 50 s of a 100 s simulation. Fig 7 shows three 2D slices through the parameter space (with the third parameter held constant at its global minimum). We see that the model’s beta power responded to all three parameters, and that a minimum existed. The sweep also revealed a complex underlying landscape with flat regions, nonlinearities, and local minima, which may prove difficult for optimization algorithms to navigate.

Fig 7 only shows three 2D slices through 3D volumetric data; there are other complex interactions which are not seen in these planes. Most importantly, however, the parameter sweep revealed that the BGTCS has a global minimum. Next, we tested the Bayesian ADC’s ability to efficiently locate the global minimum in this complex landscape.

### Individual runs

**Fig 8:**
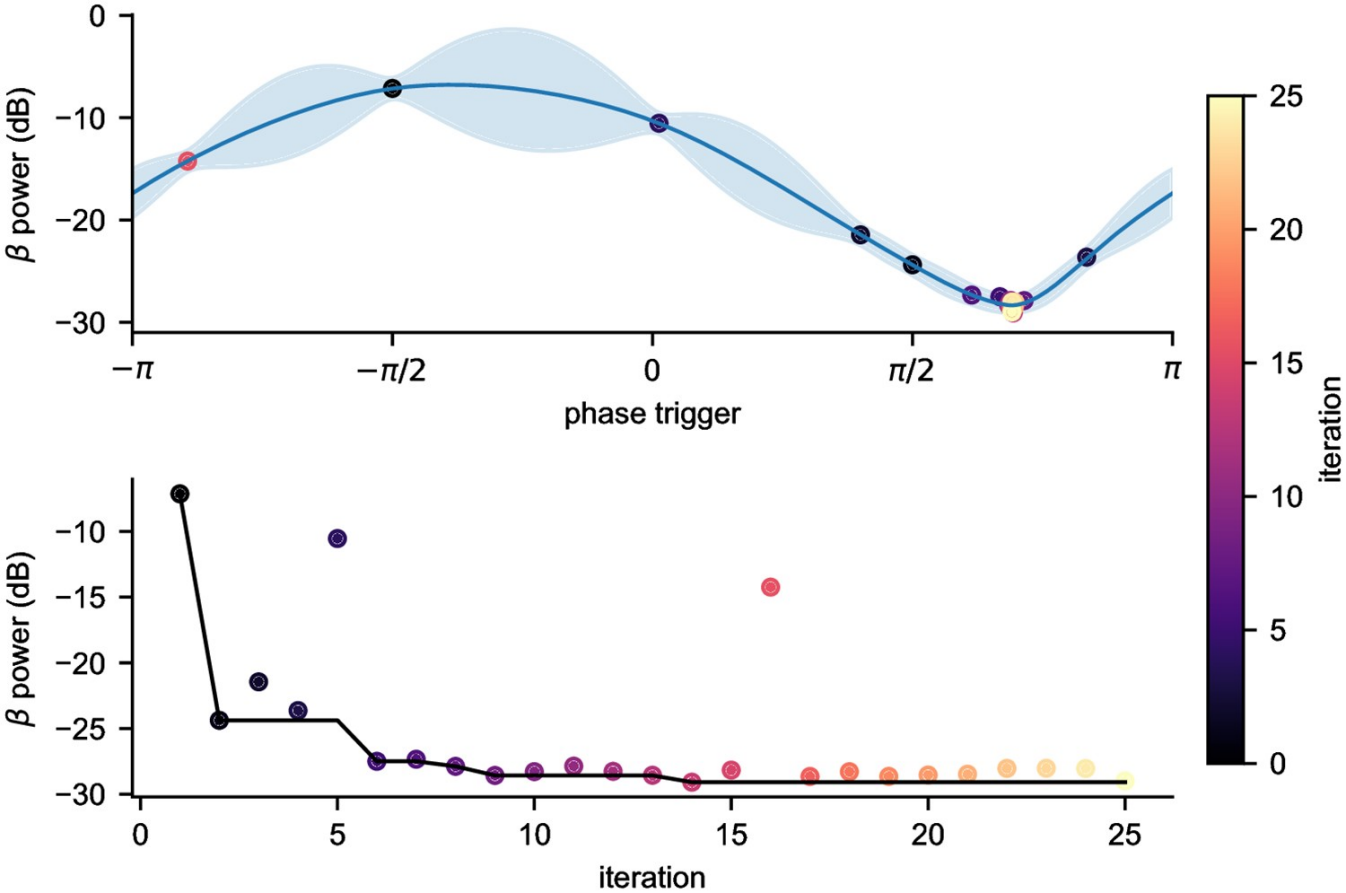
Bayesian ADC optimizing stimulus phase trigger. Example 1D optimization of stimulus phase trigger. The simulation was run for 25 iterations in which Bayesian optimization was used to select the stimulus phase trigger while holding stimulus amplitude and power threshold constant (2.37 mA, -28.6 dB). (top) Gaussian process built from observations. (bottom) Power as a function of iteration, and minimum value found. The color of each dot represents the iteration at which each parameter setting was visited during the simulation.

After having verified the existence of a global minima, we ran the Bayesian ADC in all 7 parameter combinations. In the 1 and 2D cases, the variable(s) not being optimized over were fixed at their global minimum. Fig 8 shows a 1D example of the Bayesian ADC optimizing the stimulus phase trigger, with stimulus amplitude and power threshold held constant. By the 6th function evaluation, the Bayesian ADC was already sampling near the optimal stimulus phase. The Bayesian ADC was able to build an accurate representation of the BGTCS’ response to stimulation in relatively few function evaluations. The ADC took few exploratory steps, and did so to optimally cover the space and gather information about the underlying function. In this example, we see that at function evaluation 16, the Bayesian ADC chose to explore near −*π*, before returning to the optimal region around 3*π*/4.

### Empirical analyses

Finally, we empirically analyzed the Bayesian ADC, and compared the BayesOpt outer loop’s performance to other optimization strategies. We chose to compare to two types of algorithms: gradient-approximating algorithms, such as the Nelder-Mead (NM) simplex [43], and global algorithms such as DIRect [44]. Each algorithm was bounded on the same interval, and initial conditions were selected uniformly at random. We selected NM because we expected it to outperform most other gradient-approximating algorithms, most of which are not robust to noise. The NM approximates the gradient using a simplex, whose vertices are often far enough apart to return the correct search direction, even in the presence of noise. We selected DIRect due to its ability to quickly blanket the search space.

#### Approaching the minimum

**Fig 9:**
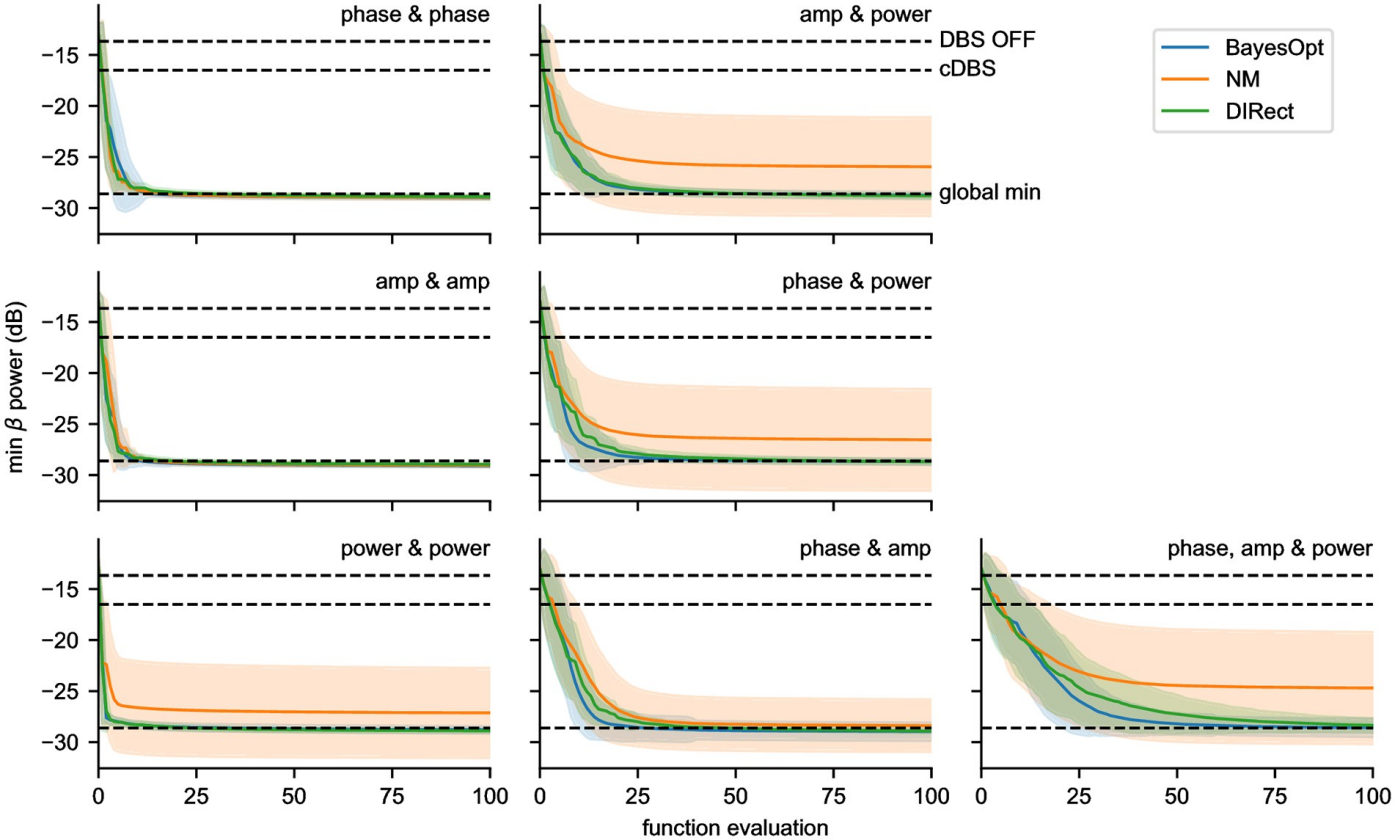
Minimum beta power found by each algorithm as a function of iteration. BayesOpt (blue) is compared against the Nelder-Mead (orange) and DIRect (green) algorithms, with the shaded region indicating the standard deviation. Each algorithm was run 1000 times in all 7 parameter combinations, and compared for their ability to find the global minimum in as few function evaluations as possible. BayesOpt and DIRect perform comparably in all cases, while NM falls behind in cases where power threshold is optimized. The dotted lines represent the global minimum beta power, as well as the beta power with DBS OFF and cDBS for comparison.

We first compared the algorithms ability to find the global minimum in the fewest number of function evaluations. Each algorithm was run 1000 times, and the mean and standard deviation of the minimum beta power found after each evaluation are reported in Fig 9. BayesOpt and DIRect performed equally well in all cases, both were able to find the global minimum robustly, and both approached the global minimum at approximately the same rate. Conversely, NM had more difficulty in reliably finding the global minimum. In 1D, the NM performed comparably in optimizing phase trigger and stimulus amplitude, but was unable to reliably find the global minimum in power threshold. In 2D, the NM performed comparably in the phase & amplitude case, but fell short in the other two cases, as well as in the 3D case.

#### Staying at the minimum

**Fig 10:**
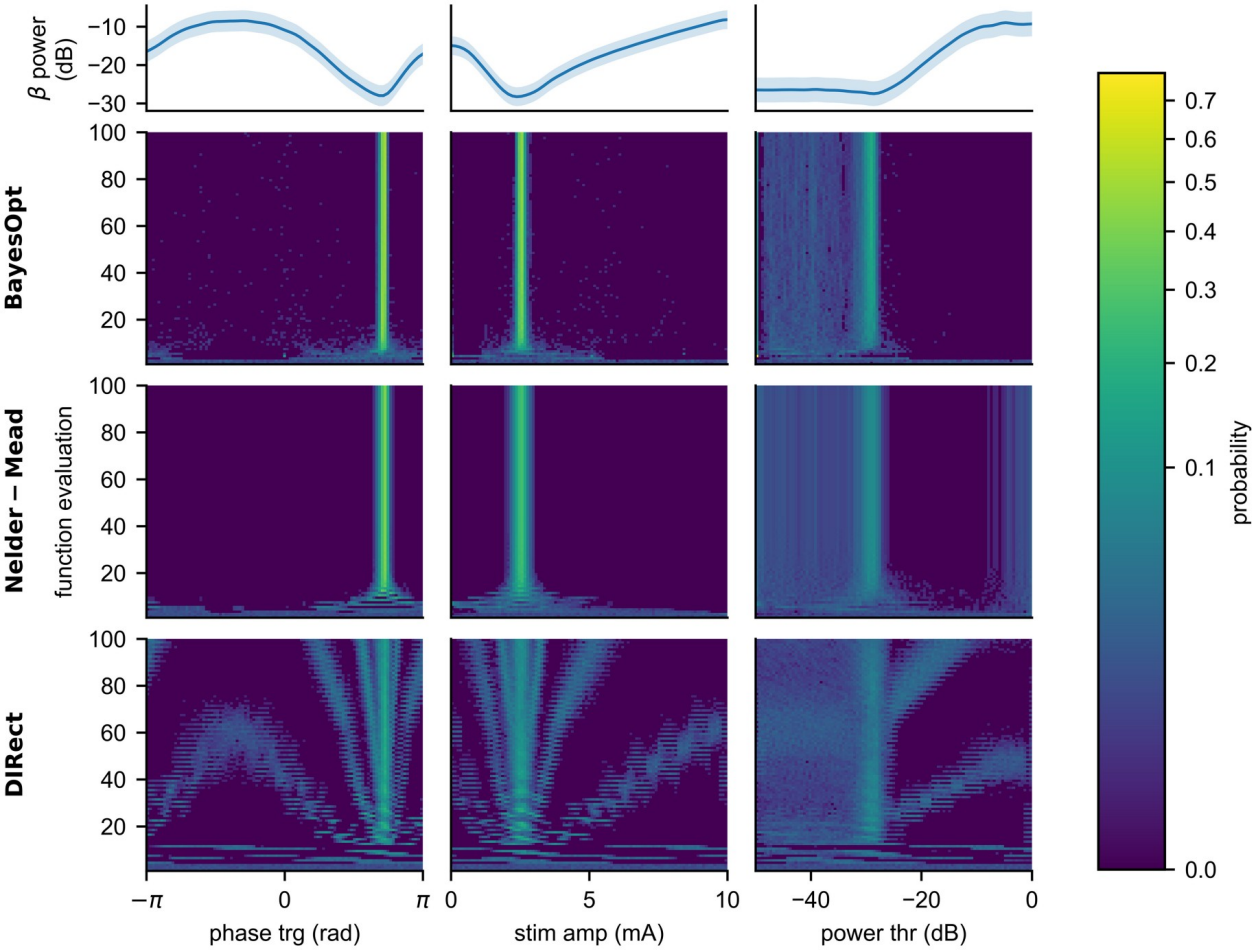
Histograms of the parameters selected by each algorithm in 1D. Histograms of the parameters selected by each algorithm (rows 2-4) over 1000 trials of 100 function evaluations are show, as well as the underlying response surfaces (top row). Each row shows the sampling patterns of an algorithm as it attempted to minimize beta power in each of the 1D cases (columns). BayesOpt clustered most tightly on the optimum parameter values in all cases. The NM algorithm explored the space the least and was easily trapped in flat regions or in a local minimum. The DIRect algorithm continually explored the space, and never transitioned to exploitation.

While both BayesOpt and DIRect were able to reliably find the global minimum, their sampling patterns differed greatly. To illustrate this difference, and compare to NM, Fig 10 shows histograms of the parameters chosen by each algorithm at each iteration over 1000 trials in the 1D cases, as well as the underlying 1D response surfaces. We see that each algorithm approached the optimal parameter values differently. BayesOpt clustered most tightly on the optimal values, followed by NM, while the DIRect algorithm continued to explore throughout the simulation. As a gradient-approximating method, NM exploited well, but explored poorly, and was easily trapped by local minima and flat regions. Conversely, as a global search algorithm, DIRect was able to find the global minimum quickly and efficiently. However, it could not transition from exploration to exploitation, and so the algorithm continued to explore the space throughout the simulation. BayesOpt balanced exploration and exploitation; the algorithm exploited well, as the parameter values quickly clusted on the optimum, more tightly than either NM or DIRect. Additionally, we can see the global exploration steps that BayesOpt took throughout the simulation, allowing it to build a model of the entire space to ensure that it found the global minimum.

#### Regret

**Fig 11:**
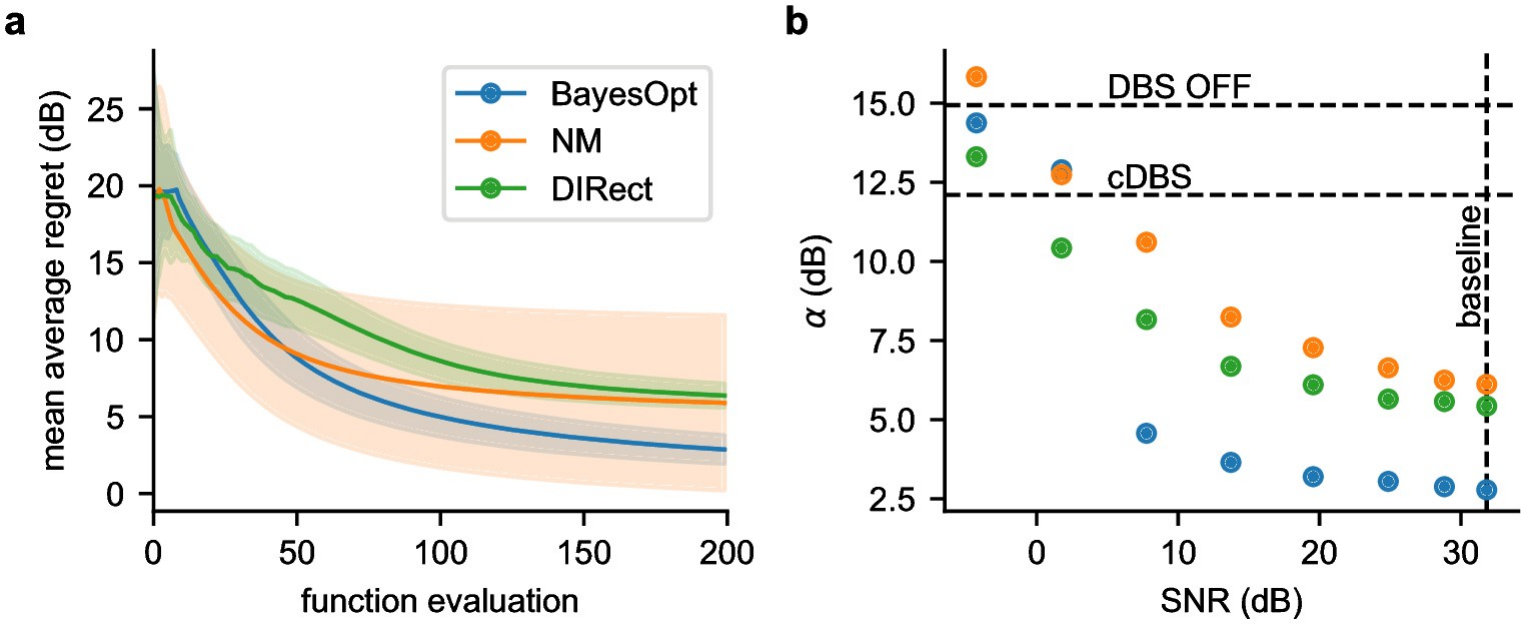
Mean average regret and noise tolerance. (a) The mean of the average regret (*R*_*T*_/*T*) across 1000 trials for each algorithm in the 3D case. BayesOpt asymptotes to the lowest regret, while NM asymptotes fastest but to higher regret. (b) Asymptotic constant, *α*, under increasingly noisy conditions. As the SNR degrades, each algorithms’ asymptotic performance deteriorates. BayesOpt continues to outperform the other algorithms at moderate to high SNRs and performs similarly at poor SNRs. The horizontal dotted lines indicate the regret incurred with DBS OFF and cDBS, while the vertical line represents the baseline SNR.

A natural performance metric that encapsulates both exploration and exploitation properties is the average cumulative regret; i.e. the loss in reward due to not knowing the global minimum before hand. Whereas the minimum found as a function of each iteration (Fig 9) shows how quickly the algorithm found a minimum, the average cumulative regret quantifies both how quickly an algorithm finds the global minimum, and how well it exploits it. At each iteration, we incur instantaneous regret *r*_*t*_ = *f*(*θ*_*t*_) − *f*(θ*), where *f*(θ*) is the function value at the best parameters, θ*. The *cumulative regret* after *T* iterations is $R_{T}=\sum_{t=1}^{T}r_{t}$, and a desirable asymptotic property of an optimization algorithm is to be *no-regret*: lim_*T*→∞_ *R*_*T*_/*T* = 0 [42]. Therefore, an algorithm who’s average cumulative regret asymptotes to a lower value can be considered superior. The performance of each algorithm was quantized by fitting an exponential decay function of the form *R*_*T*_/*T* = *α* + *T*_0_ exp(−*T*/*τ*), which asymptotes to *α* with a time constant of *τ*. Fig 11a shows the mean average regret (the mean across 1000 trials of the average regret up to iteration *T*), and Table 1 reports the asymptotes and time constants fit to each algorithm in the 3D case. BayesOpt had the lowest asymptote, reflecting the algorithms ability to reliably find and stay at the global minimum. However, NM had the shortest time constant, reflecting its ability to quickly descend towards a minimum (be it local or global).

**Table 1:** Asymptote and time constant of each algorithm in the 3D case.

Algorithm	*α*	*τ*
BayesOpt	2.78 ± 0.02	45.7 ± 0.26
DIRect	5.43 ± 0.03	68.3 ± 0.45
Nelder-Mead	6.12 ± 0.04	32.8 ± 0.52

#### Noise tolerance

Finally, we quantified the performance of the three different algorithms under increasingly noisy conditions. To test each algorithm, we added normally distributed noise $\epsilon ∼N(0,\sigma^{2})$ to the beta power measurement passed to the optimization algorithms. To quantify the signal-to-noise ratio (SNR), we estimated the signal amplitude as the standard deviation of beta power across the search space, and the baseline noise amplitude as the standard deviation of repeated measures. For each algorithm, we ran 500 trials under increasing noise, and estimated the asymptotic regret, *α*, shown in Fig 11b. As expected, we see that all three algorithms’ asymptotes increase as the SNR decreases from baseline, with BayesOpt continuing to outperforming the other algorithms at moderate to high SNRs. As the SNR degrades below 5 dB, BayesOpt’s asymptote sharply increases, putting it in line with the other algorithms. At this point, each of the three algorithms’ asymptotic performance is comparable to cDBS. As it takes many iterations to reach the asymptote, a static solution (such as cDBS) may be preferable under noisy conditions.

### In summary

- NM is able to descend faster than both BayesOpt and DIRect, but is unable to reliably find the global minimum;
- DIRect reliably finds the global minimum, but continues to explore and so has high regret;
- BayesOpt reliably finds the global minimum, and has the lowest regret;
- BayesOpt is more robust to noise.

## Discussion

In this paper, we present a Bayesian adaptive dual control algorithm for optimizing DBS stimulation parameters to suppress pathological oscillations. The Bayesian ADC was tested in a computational model of the basal ganglia-thalamocortical system, which exhibited an emergent oscillation in the dopamine-depleted state that was suppressed by cDBS. The use of the BGTCS model (as opposed to a simple coupled oscillator model), provides more directly translatable results, and allows us to draw several physiologically relevant conclusions about how closed-loop stimulation might function in a real system. The Bayesian ADC algorithm was composed of two pieces: an inner feedback controller and an outer parameter optimization loop. The inner feedback loop was shown to have the potential to be more effective at suppressing the model’s pathological oscillation than cDBS, but was sensitive to parameter changes. The outer BayesOpt parameter adjustment loop was shown to be more efficient in selecting the optimal parameters for the inner loop than other optimization methods. While the focus of this paper was on suppressing an oscillation seen in the dopamine-depleted state of a computational model, the Bayesian ADC is general both for the feedback signal optimized by the outer loop and for the stimulator employed by the inner loop.

### Biological insights into closed-loop stimulation

Through examination of the optimization landscape (Fig 7), we can draw several key insights regarding the nature of closed-loop stimulation in the context of a biological system, and relate these results to other studies in the field.

#### Phasic stimulation

The use of phasic stimulation to suppress pathological oscillations has been previously proposed and tested, both in computational models [9, 10, 45–47] and in human tremor patients [11, 12]. While the mechanisms of phasic stimulation are well understood in the context of simple models of coupled oscillators [45–47], the precise mechanisms of phasic stimulation in the context of the brain is not well understood. The computational model used to simulate the response of the basal ganglia to phasic stimulation displayed several interesting features which may provide insight into the potential mechanisms of phasic stimulation.

We hypothesize that oscillations can be generated by two separate mechanisms in the basal ganglia: 1) under pathological conditions neurons start to fire in bursts generating beta rhythms, or 2) an inhibitory and excitatory reciprocally connected neuronal populations, such as the STN and GPe, start generating oscillations that have matched resonances and produce beta oscillations. These oscillations could be suppressed in a number of manners. First, phasic stimulation could simply suppress the firing rate of the neurons. However, in this model the firing rates are not significantly affected. Second, phasic stimulation could alter the temporal spiking relationship between connected neurons, and through spike-timing dependent plasticity (STDP), alter the strength of synaptic connections within and between oscillating structures [12, 48]. However, as STDP is not incorporated in this model we can rule out this effect in the model presented here. Third, phasic stimulation could interact with individual spike timing within an oscillating population. By stimulating at certain phases, the population can be desynchronized so that they no longer produce burst dynamics [10]. However, as the BGTCS does not model individual neurons, we can rule out this effect in this model as well. Fourth, phasic stimulation could simply mask the oscillation through destructive interference by applying stimulation to excite neurons out-of-phase, when they are most suppressed. Finally, the stimulus can interact with the instantaneous frequency of an oscillator, effectively moving the system closer to, or further from, peak resonance [49, 50]. This is the mechanism observed in models of coupled oscillators.

This leaves us with the task of determining if the oscillations are being suppressed by destructive interference or by modulating the resonance dynamics. First, we note that while we are stimulating and recording in the STN and GP respectively, the model’s synaptic delay (1 ms) between the STN and GP is much shorter than the oscillation period (34.5 ms); as such the phase difference between these structures is negligible. Upon closer inspection, we notice several features which support the modulation of resonance dynamics. First, we notice that the optimal stimulus phase for suppression (2.24 rad) occurs during the downward phase of the oscillation (here 0 rad and ±π rad aligns to the peak and trough of the oscillation, respectively), while the optimal phase for enhancement occurs ±π rad out, during the rising phase of the oscillation. Furthermore, we see that the optimal stimulus phase for suppression depends on stimulus amplitude: As stimulus amplitude increases, the optimal stimulus phase precesses towards peak depolarization (from 2.24 rad to 1.57 rad). The optimal phase for enhancement does not depend upon the stimulus amplitude.

If the oscillations were suppressed/enhanced by destructive/constructive interference, we would expect that 1) the optimal phase for suppression/enhancement would align with the trough/peak (±π /0 rad), and 2) we would not expect the optimal phase to change with the amplitude of the stimulation. This in our opinion rules out destructive interference. We hypothesize that instead the stimulation effectively changes the resonance properties of the reciprocal excitatory/inhibitory coupling between the STN and GPe. Stimulation during the falling phase of the oscillation results in a transient phase delay, moving the STN/GPe away from peak resonance. The magnitude of this phase delay is amplitude dependent, and can even become a phase advance at large amplitudes, explaining the amplitude-dependence of the optimal suppression phase. Conversely, stimulation during the rising phase results in a transient phase advance, resulting in stronger resonance between the two structures [45]. This prediction is one that can be tested experimentally, and might provide insight into the mechanism of beta suppression.

#### Power thresholded stimulation

There are also several studies which use beta power to turn stimulation on or off, but to our knowledge, no one has tried to combine phase and power based stimulation. Power thresholded stimulation has been implemented in two different ways: (a) a threshold is used to turn stimulation on or off [7, 8], and (b) stimulation amplitude is ramped up or down based on either beta [51, 52] or tremor power [53]. The idea behind a beta threshold is to turn off/down stimulation when it’s not needed, e.g. when beta is low. Here, we see a straightfoward effect of the power threshold on beta power: given the optimal stimulus phase and amplitude, the model’s beta power is drawn down to the power threshold, until reaching the model minimum. Therefore, a power threshold parameter could be explicitly used as a “beta thermostat”, allowing for the avoidance of side-effects that could be induced by over-suppressing beta power.

### Advantages

The Bayesian ADC’s key advantages stem from fitting a Gaussian process (GP) to data, and then using the GP to intelligently sample, explicitly balancing exploration and exploitation to find the global minimum. Through fitting the GP, BayesOpt is able to learn and account for both the length scale of the parameters as well as the noise level, and is among the most efficient algorithms in terms of number of function evaluations required to find the minimum. The Bayesian ADC is also able to balance exploration and exploitation: it is able to find the minimum quickly and exploit it, but continues to explore intelligently to ensure that it has arrived at the global minimum. Gradient-approximating methods (such as NM) can quickly descend towards a minimum, but are unable to explore globally, are sensitive to initial conditions, and are vulnerable to becoming trapped in local minima or wandering around flat regions. Global exploration methods (such as DIRect), do not rely on gradients and can quickly find the global minimum. However, such algorithms are often purely exploratory, and never transition to exploitation.

When trying to optimize stimulation parameter settings, balancing exploration and exploitation is critical. We need to approach the minimum as quickly as possible, but also avoid local minima while preventing unnecessary exploration, as the patient is likely to have little tolerance for wildly varying stimulation parameters. BayesOpt provides a framework for balancing exploration and exploitation in a way that most other algorithms do not.

Of course, selecting the optimal balance between exploration and exploitation is not a trivial task. All acquisition functions have a hyperparameter which controls the exploration/exploitation tradeoff. The GP-UCB algorithm we emplyed is *no regret* in the limit as *N* → ∞ with *ν* = 1. However, we are less interested in achieving 0 regret than we are with achieving a low regret quickly. Thus, a smaller *ν* should be chosen, such as *ν* = 0.25, to encourage exploitation. Furthermore, this hyperparameter could be adapted over time: if the patient feels like too many exploratory settings are being chosen, *ν* could be decreased.

### Limitations

The Bayesian ADC is not without its limitations. First and foremost, because Bayesian optimization relies on calculating and inverting the covariance matrix of the inputs, the complexity grows as *O*(*n*^3^), where *n* is the number of observations. Therefore, the time it takes to compute the next parameter set increases as the cube of the number of samples. However, in this case, and many clinical applications, the time it takes to assess the effects of a single parameter set is relatively long (seconds in this model, minutes in the clinic, or hours or even days at home). Therefore, as long as it takes less time to compute the next parameter set than it does to evaluate a parameter set, this will not be an issue.

Additionally, the Bayesian ADC assumes the existence of a static response surface, although this need not be the case. If the patient’s response to stimulation is changing over the course of the measurements, the Bayesian ADC will not converge. However, if the time-course of this change is long relative to the time-course of the measurements, this could be overcome. Furthermore, instead of using all previous observations, we could limit the algorithm to use only the most recent *N*, thereby allowing the algorithm to “forget”, forcing it to re-explore changing areas. This “forgetting” strategy could be used to solve the aforementioned complexity problem as well. Finally, neural networks (NNs) could be used to estimate the GP, which would address both the scalability problem (becoming linear in *n*, instead of cubic), and the stationarity problem (as NNs naturally “forget” training data far in the past) [54, 55].

### Generalizability

The Bayesian ADC framework we present here has broad applicability for tuning stimulation parameters across diseases and devices. At its heart, the Bayesian ADC framework is simply a method for efficiently optimizing the parameters of a controller using some feedback signal. Both the inner loop and the feedback signal can be designed to fit the problem at hand.

#### Inner loop

In our Bayesian ADC, we used a phase/power feedback controller for the inner loop. However, any controller, closed- or open-loop, could be used for the inner loop. This means that the Bayesian ADC can be used to tune stimulation parameters for any stimulator, from current open-loop cDBS to state-of-the-art closed-loop algorithms.

#### Feedback signals and objective functions

The Bayesian ADC can be used to tune stimulation parameters for any disease by selecting the appropriate feedback signal (or signals) and defining an objective function over those feedback signals. In our case, we chose to minimize the power of the beta oscillation measured from the BGTCS. However, there is concrete evidence that not all exaggerated beta oscillations are pathological, and that it may serve as a non-exact biomarker for PD severity [56–60]. Other neurophysiological biomarkers have been proposed for PD, including phase-amplitude coupling (PAC) [21, 61, 62] and evoked compound action potentials (ECAPs) [63], to name a few. Additionally, kinematic feedback signals could be incorporated, such as quantitative measures of tremor, rigidity, bradykinesia, or other symptoms. The feedback signal need not be physiological, it could be a qualitative behavioral or quality of life metric, measured by the patient, clinician, or family member.

Finally, side effects could be taken into account by allowing the patient to self-report the severity and frequency of side effects. Our objective function would then become a weighted combination of the feedback signal as well as side effect. By incurring a penalty whenever side effect occurs, the Bayesian ADC can learn to avoid those parameters (even if they positively affected other feedback signals). However, as with any multi-objective optimization problem, we are then left with the task of assigning relative weights to the individual components.

#### Diseases

The Bayesian ADC is not limited to PD, but is generalizable to any disease with a parameterized stimulator. For example, the Bayesian ADC could be easily adapted to optimize DBS parameters to treat essential tremor. In this case, the feedback signal could be the tremor power measured from a wrist-mounted accelerometer. The inner loop could be a simple continuous stimulator, a phase/power feedback stimulator, or any parameterized stimulator. Indeed, recent work has indicated that using kinematic biomarkers (specifically triggering stimulation off the power of the patient’s tremor) could improve the efficacy of closed-loop DBS [53].

#### Discrete and categorical parameters

The Bayesian ADC is not limited to optimizing continuous parameters, but can also handle discrete and parameters. Discrete parameters are those that possess an inherent *ordering* or *structure*, such that the effects of two “nearby” parameters can be expected to be more similar than two “distant” parameters. For example, in the case of electrode configuration, stimulating through contact 1 on a traditional four contact DBS lead can be considered to be *more similar* to stimulating through contact 2 than through 3, as it is more likely to activate similar or overlapping neuronal populations. Thus, we need only come up with a numerical encoding of the discrete parameters, which could then be used to compute the kernel between any two parameter sets. In the above example, we might encode contact 1 = 1, contact 2 = 2, etc. We would then be able to run these discrete parameters through the kernel function, and we would see that *k*(1, 2) > *k*(1, 3). We would then be left with the problem of learning a suitable length constant in the electrode contact dimension which encapsulates the spatial relation between contacts. In the case of categorical parameters with no underlying structure, a multi-armed bandit solution should be implemented instead [64].

### Conclusion

In this paper, we present a Bayesian adaptive dual controller for the suppression of pathological oscillations. The Bayesian ADC was shown to perform well in a computational model of Parkinson’s disease for selecting the optimal parameters to reduce the oscillation power. The Bayesian ADC was composed of two parts, an inner feedback stimulator, and an outer BayesOpt parameter tuning loop. As compared to other algorithms, BayesOpt was able to efficiently tune stimulation parameters, explicitly balancing exploration and exploitation to find the optimal settings in as few function evaluations as possible. Finally, the Bayesian ADC is generalizable, both across diseases and stimulator designs.
